# Differences in fungi present in induced sputum samples from asthma patients and non-atopic controls: a community based case control study

**DOI:** 10.1186/1471-2334-13-69

**Published:** 2013-02-05

**Authors:** Hugo Cornelis van Woerden, Clive Gregory, Richard Brown, Julian Roberto Marchesi, Bastiaan Hoogendoorn, Ian Price Matthews

**Affiliations:** 1 Institute of Primary Care & Public Health, Cardiff University School of Medicine, Neuadd Meirionnydd, Heath Park, Cardiff CF14 4YS, UK; 2 Cardiff School of Bioscience, Main Building, Museum Avenue, Cardiff, CF10 3AT, UK

**Keywords:** Asthma, Sputum, Fungi, Case–control study

## Abstract

**Background:**

There is emerging evidence for the presence of an extensive microbiota in human lungs. It is not known whether variations in the prevalence of species of microbiota in the lungs may have aetiological significance in respiratory conditions such as asthma. The aim of the study was to undertake semi-quantitative analysis of the differences in fungal species in pooled sputum samples from asthma patients and controls.

**Methods:**

Induced sputum samples were collected in a case control study of asthma patients and control subjects drawn from the community in Wandsworth, London. Samples from both groups were pooled and then tested for eukaryotes. DNA was amplified using standard PCR techniques, followed by pyrosequencing and comparison of reads to databases of known sequences to determine in a semi-quantitative way the percentage of DNA from known species in each of the two pooled samples.

**Results:**

A total of 136 fungal species were identified in the induced sputum samples, with 90 species more common in asthma patients and 46 species more common in control subjects. *Psathyrella candolleana, Malassezia pachydermatis, Termitomyces clypeatus* and *Grifola sordulenta* showed a higher percentage of reads in the sputum of asthma patients and *Eremothecium sinecaudum, Systenostrema alba, Cladosporium cladosporioides* and *Vanderwaltozyma polyspora* showed a higher percentage of reads in the sputum of control subjects. A statistically significant difference in the pattern of fungi that were present in the respective samples was demonstrated using the Phylogenetic (P) test (P < 0.0001).

**Conclusion:**

This study is novel in providing evidence for the widespread nature of fungi in the sputum of healthy and asthmatic individuals. Differences in the pattern of fungi present in asthma patients and controls merit further investigation. Of particular interest was the presence of *Malassezia pachydermatis*, which is known to be associated with atopic dermatitis.

## Background

The human lung has a surface area of around 50 m^2^[1] and is in contact with more than 15,000 litres of air each day [2]. At each breath around 5,000 particles of dust are inhaled [3]. On average, the dust in the earth’s atmosphere contains 10,000 to 100,000 organisms per gram of dust [4], some of which is in the ‘respirable dust’ fraction, consisting of particles smaller than 5 μm [5]. This extensive exposure to the environment means that the lungs are a common portal for infection by viruses, bacteria, fungi, protozoa and other infectious agents.

Historically, healthy lungs were believed to be free of bacteria and that during infection organisms “gain a foothold in the normally sterile lung tissue” [6]. However, there is increasing evidence that microbiota are present even in healthy lungs [7]. This finding raises the possibility of a potential overlap between pathogenic and commensal microbiota in the respiratory tract.

There is relatively little literature examining microbiota in human lungs. Tunney et al. [8] showed that approximately 50% of healthy individuals harbour between 1000 and 10,000 culturable anaerobic bacteria per ml of induced sputum A range of microbial species were also found in induced sputum at low numbers in another study which examined sputum from healthy subjects [9]. One previous study has been identified which used metagenomic culture independent genomic techniques and demonstrated that microbial communities in asthmatic airways were disordered, with pathogenic *Proteobacteria* more frequently found in the bronchi of asthmatics patients than in controls [10].

The current study further examines the role of atypical microbiota in respiratory disease. The study used molecular techniques to identify eukaryote species that were present in induced sputum samples taken from asthma patients and controls, living in Wandsworth, London. The aim of the study was to undertake semi-quantitative analysis of the differences in fungal species present in pooled sputum samples from asthma patients and controls.

## Methods

### Study population

The study protocol was approved by Camden and Islington community local research ethics committee (ref 08/H0722/540). All patients participating in the study supplied informed consent. This case control study from which the induced sputum samples were drawn has previously been described. Further information on the characteristics of the subjects in this study has been provided in that paper [11]. In summary, participants were residents of Wandsworth, London, and were primarily identified from the patient registers of two GP practices. Asthma patients were defined as those individuals who had a current diagnosis of asthma, for example, by being on the GP practice asthma register. Most of the asthma patients were on inhaled corticosteroids. Non-atopic controls were defined as individuals who on questioning did not report having current or previous asthma, eczema or hay fever. All participants competed a published questionnaire [12] to assess the risk of mould in the home. The questionnaire contained four questions: Is there any visible mould growth on your house? Is there any odour of mould or cellar-like musty air in your house? Is there any moisture stains in your house? Is there any water/moisture damage in your house?

### Sputum collection and DNA extraction

Participants inhaled isotonic saline via an ultrasonic nebuliser. Globules of sputum were coughed up into petri dishes, spread on microscope slides and stained for microscopic examination. Approximately 5 mm^2^ areas were excised from each microscope slide. The samples were combined to yield two pooled samples for subsequent DNA extraction and PCR: asthma patients and control subjects. (A sample from one asthma patient was inadvertently included in the control set). DNA was subsequently extracted using the Zymo research pinpoint system (Zymo Research, Irvine, Ca) in accordance with manufacturer’s instructions. The samples were taken from 30 asthma patients and 13 non-atopic control subjects involved in the case control study.

### Pyrosequencing of extracted DNA and statistical analysis

Extracted DNA was amplified using a PCR protocol for the partial 18S rRNA gene using the primer pair (Euk1a (5’ CTG GTT GAT CCT GCC AG 3’) and Euk516r (5’ ACC AGA CTT GCC CTC C 3’)) in accordance with previously described protocols [13,14]. The two pooled extract amplicons, from asthma patients and from controls, were sequenced using a 454 pyrosequencer by Research and Testing Inc, Lubbock, Texas, USA. DNA sequences were compared to the SILVA database of known eukaryotic 18S rRNA gene sequences to determine in a semi-quantitative way the proportional distribution in each of the two samples.

The difference between the pattern of fungal species in each of the two pooled samples was compared using Unifrac [15,16]. This online software uses phylogenetic information to test whether or not two environments are significantly different. The software estimates the similarity between communities by measuring the number of changes that would be required to explain the differences in the distribution of sequences between the two environments.

## Results

### Study population and presence of mould in the home

Patients had a mean age of 41.6 years (SD 14.9, range 18–65 years) and control participants a mean age of 35.7 years (SD 12.8, range 24 – 58 years). The patient group was 40% male and the control group was 46% male.

A positive answer to at least one of the four questions regarding possible mould in the home was recorded in 30% (9/30) asthma patients and 15.4% (2/13) of control subjects. Due to the small sample size, this relatively large difference was not statistically significant.

### Analysis of pyrosequencing data

The differences based on the percent of total DNA reads of in the pooled samples from asthma patients and non-atopic controls are shown in Tables 1 and 2. A statistically significant difference in the pattern of fungi that were present in the respective samples was demonstrated using the Phylogenetic (P) test (P <0.0001).

**Table 1:** Fungi that were more common in asthma patients than in control participants (difference in percent of DNA reads in descending order)

**Fungal species**	**Control participants**	**Asthma patients**	**Difference**
*Psathyrella candolleana*	0.000	27.294	27.294
*Malassezia pachydermatis*	0.000	21.651	21.651
*Termitomyces clypeatus*	0.000	7.071	7.071
*Grifola sordulenta*	0.000	4.489	4.489
*Pycnoporus sp*	0.000	2.938	2.938
*Phlebiopsis gigantean*	0.000	2.932	2.932
*Dichostereum pallescens*	0.000	2.746	2.746
*Peniophorella praetermissa*	0.168	2.617	2.449
*Aspergillus zonatus*	0.016	1.959	1.942
*Acanthophysium cerussatum*	0.000	1.825	1.825
*Pleurotus ostreatus*	0.000	1.615	1.615
*Candelabrochaete africana*	0.016	1.556	1.540
*Basidiobolus ranarum*	0.000	1.510	1.510
*Tapinella atrotomentosa*	0.000	1.487	1.487
*Pleurocybella porrigens*	0.000	1.399	1.399
*Debaryomyces hansenii*	0.000	1.294	1.294
*Collybia tuberosa*	0.000	1.282	1.282
*Galerina atkinsoniana*	0.000	1.230	1.230
*Punctularia strigosozonata*	0.000	1.148	1.148
*Elderia arenivaga*	0.000	1.143	1.143
*Pseudoarmillariella ectypoides*	0.000	0.834	0.834
*Pulcherricium caeruleum*	0.000	0.665	0.665
*Tilletia goloskokovii*	0.000	0.618	0.618
*Cerrena sp*	0.000	0.600	0.600
*Serpula lacrymans*	0.000	0.571	0.571
*Bondarcevomyces taxi*	0.000	0.554	0.554
*Resinicium bicolor*	0.000	0.519	0.519
*Cortinarius sodagnitus*	0.000	0.414	0.414
*Trichaptum abietinum*	0.000	0.315	0.315
*Chamaeota sinica*	0.000	0.262	0.262
*Peziza vesiculosa*	0.000	0.239	0.239
*Pterula echo*	0.000	0.204	0.204
*Laccocephalum mylittae*	0.000	0.198	0.198
*Coprinopsis cinerea*	0.000	0.198	0.198
*Exidiopsis calcea*	0.000	0.187	0.187
*Dioszegia fristingensis*	0.000	0.157	0.157
*Inonotus baumii*	0.060	0.210	0.150
*Hydnochaete olivacea*	0.000	0.134	0.134
*Derxomyces boekhoutii*	0.000	0.128	0.128
*Xeromphalina campanella*	0.000	0.117	0.117
*Pulchromyces fimicola*	0.000	0.111	0.111
*Aspergillus oryzae*	0.000	0.099	0.099
*Entoloma prunuloides*	0.000	0.099	0.099
*Dioszegia zsoltii*	0.000	0.093	0.093
*Basidiobolus haptosporus*	0.000	0.087	0.087
*Saccharomycopsis fibuligera*	0.000	0.087	0.087
*Galiella rufa*	0.000	0.082	0.082
*Derxomyces simaoensis*	0.000	0.070	0.070
*Mycoclelandia arenacea*	0.000	0.064	0.064
*Steccherinum fimbriatum*	0.000	0.064	0.064
*Austropaxillus sp*	0.000	0.058	0.058
*Meyerozyma guilliermondii*	0.000	0.058	0.058
*Volvariella caesiotincta*	0.000	0.058	0.058
*Galerina marginata*	0.000	0.052	0.052
*Occultifur externus*	0.000	0.052	0.052
*Hericium americanum*	0.000	0.047	0.047
*Penicillium commune*	0.000	0.041	0.041
*Volvopluteus earlei*	0.000	0.041	0.041
*Gymnopus dryophilus*	0.000	0.029	0.029
*Aspergillus terreus*	0.000	0.023	0.023
*Tritirachium sp*	0.000	0.023	0.023
*Gloiocephala aquatic*	0.000	0.023	0.023
*Tricholoma matsutake*	0.000	0.023	0.023
*Exidia uvapsassa*	0.000	0.017	0.017
*Rhodocollybia maculate*	0.000	0.017	0.017
*Lentinus sp*	0.000	0.017	0.017
*Teratosphaeria acidotherma*	0.076	0.093	0.017
*Taphrina deformans*	0.000	0.012	0.012
*Antrodia vaillantii*	0.000	0.012	0.012
*Aspergillus penicillioides*	0.000	0.012	0.012
*Passalora vaginae*	0.000	0.012	0.012
*Malassezia furfur*	0.000	0.012	0.012
*Candida sp*	0.000	0.012	0.012
*Piromyces sp*	0.000	0.012	0.012
*Paxillus vernalis*	0.000	0.012	0.012
*Derxomyces mrakii*	0.000	0.012	0.012
*Lasiodiplodia gonubiensis*	0.000	0.012	0.012
*Teratosphaeria ohnowa*	0.005	0.012	0.006
*Diversispora celata*	0.000	0.006	0.006
*Geomyces destructans*	0.000	0.006	0.006
*Coprinopsis sp*	0.000	0.006	0.006
*Chlamydosauromyces punctatus*	0.000	0.006	0.006
*Mortierella minutissima*	0.000	0.006	0.006
*Saccobolus dilutellus*	0.000	0.006	0.006
*Thanatephorus fusisporus*	0.000	0.006	0.006
*Boletellus shichianus*	0.000	0.006	0.006
*Puccinia poarum*	0.000	0.006	0.006
*Mallocybe dulcamara*	0.000	0.006	0.006
*Coniophora marmorata*	0.000	0.006	0.006
*Trichosporon sp*	0.000	0.006	0.006

**Table 2:** Fungi that were more common in control participants than in asthma patients (difference in percent of DNA reads in descending order)

**Fungal species**	**Control participants**	**Asthma patients**	**Difference**
*Eremothecium sinecaudum*	41.319	1.026	40.293
*Systenostrema alba*	23.587	0.000	23.587
*Cladosporium cladosporioides*	14.484	0.111	14.374
*Vanderwaltozyma polyspora*	6.778	0.140	6.638
*Entophlyctis helioformis*	2.976	0.064	2.912
*Rozella allomycis*	3.009	0.198	2.811
*Protomyces macrosporus*	1.971	0.082	1.890
*Mortierella verticillata*	1.135	0.000	1.135
*Pseudotaeniolina globosa*	1.086	0.210	0.876
*Dothidea ribesia*	0.701	0.000	0.701
*Sporobolomyces yunnanensis*	0.549	0.006	0.543
*Teratosphaeria mexicana*	0.261	0.000	0.261
*Myriangium duriaei*	0.179	0.000	0.179
*Phaeobotryosphaeria visci*	0.174	0.000	0.174
*Kionochaeta sp*	0.152	0.012	0.140
*Catenulostroma chromoblastomycosum*	0.157	0.017	0.140
*Phaeobotryon mamane*	0.125	0.000	0.125
*Allomyces arbuscula*	0.125	0.000	0.125
*Schizothyrium pomi*	0.109	0.000	0.109
*Mycosphaerella endophytica*	0.103	0.000	0.103
*Penidiella columbiana*	0.098	0.000	0.098
*Aspergillus fumigatus*	0.174	0.087	0.086
*Cladosporium sp*	0.081	0.000	0.081
*Aleuria aurantia*	0.054	0.000	0.054
*Ascodesmis sphaerospora*	0.049	0.000	0.049
*Penicillium decumbens*	0.043	0.000	0.043
*Metschnikowia bicuspidata*	0.027	0.000	0.027
*Dothidea insculpta*	0.016	0.000	0.016
*Dendryphiella arenaria*	0.016	0.000	0.016
*Aigialus rhizophorae*	0.011	0.000	0.011
*Scutellospora spinosissima*	0.011	0.000	0.011
*Volvariella volvacea*	0.011	0.000	0.011
*Flammulina velutipes*	0.011	0.000	0.011
*Candida bituminiphila*	0.005	0.000	0.005
*Ascobolus carbonarius*	0.005	0.000	0.005
*Cyttaria sp*	0.005	0.000	0.005
*Halosarpheia japonica*	0.005	0.000	0.005
*Phymatotrichopsis omnivora*	0.005	0.000	0.005
*Sporobolomyces sp*	0.005	0.000	0.005
*Orphella haysii*	0.005	0.000	0.005
*Coccocarpia erythroxyli*	0.005	0.000	0.005
*Termitaria sp*	0.005	0.000	0.005
*Candida glabrata*	0.005	0.000	0.005
*Schizosaccharomyces japonicus*	0.005	0.000	0.005
*Cladochytrium sp*	0.005	0.000	0.005
*Cyttaria hookeri*	0.011	0.006	0.005

**Figure 1:**
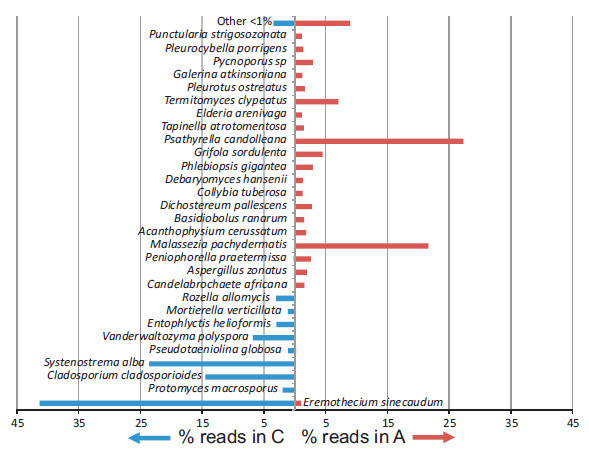
**Graph showing the percentage of pyrosequencing reads for fungal species identified in the Asthma patient (A) or control participant (C) samples (species identified for reads greater than 1%).**

A total of 136 fungal species were identified in the induced sputum samples, with 90 species more common in asthma patients and 46 species more common in control subjects, based on the percent of total DNA reads (see Figure 1). *Psathyrella candolleana, Malassezia pachydermatis, Termitomyces clypeatus* and *Grifola sordulenta* were particularly prevalent in the sputum of asthma patients and *Eremothecium sinecaudum, Systenostrema alba, Cladosporium cladosporioides* and *Vanderwaltozyma polyspora* were particularly prevalent in the sputum of control subjects. No other eukaryote species were identified in the sputum samples.

## Discussion

The range of fungal species present in both asthma patients and control subjects was larger than expected. There were also clear differences in the pattern of fungal species between asthma patients and control subjects. The fungi *Malassezia pachydermatis*, was found in patients with asthma and not the control group. This organism has a known association with atopic conditions including atopic dermatitis [17]. However, there were no other obvious associations were identified in the published literature between asthma and the other fungi found in the pooled samples from the asthma patients. Two of the fungi most commonly found in the sputum of asthma patients (*Termitomyces clypeatus* and *Psathyrella candolleana*) represent members of the basidiomycete family [18]. The latter has been found in indoor dust [19] and one can speculate that fungal spores may have been inhaled within the home. It is possible that most of the fungi identified could have come from a single individual, or a small number of individuals, whose samples were heavily colonised by fungi.

Except for *Cladosporium,* the species identified in induced sputum are not commonly found in air samples examined using standard culture techniques [20]. Analysis of air samples using molecular techniques may demonstrate that these species are commonly present in the air, but this research has not been undertaken so far. Three out of four species detected in the sputum of asthma patients were from the macromycetes group (commonly known as mushrooms). Although asthma is associated with damp environments that are affected by mould growth, we are unaware of any study that has identified an association between macromycetes and asthma. Future studies should consider analysing air samples from the homes of participants using molecular techniques, so as to take into account the presence of fungi in the ambient environment of participants.

We used universal primers for the eukaryotic 18S rRNA gene and were surprised that no eukaryotes other than fungi were identified in cases or controls. We have considered a number of potential reasons why this may be the case and the most likely was that levels of non-fungal eukaryotic DNA, present in the samples, was below the limits of detection. The PCR primers were chosen after considerable deliberation and a probeCheck test showed that they were universal and matched *Homo sapiens*’ 18S rRNA gene 100% [21]. However other potential reasons include: a genuine absence of other eukaryotes and unintended removal of DNA from other eukaryotes as part of the processing of the samples.

Individual level analysis of samples was considered, but rejected as it was anticipated that, if the samples from each individual were analysed separately, the number of eukaryotes in each sample would be below the threshold of detection. Samples for this study were therefore pooled to maximise the number of copies of each species in the pooled samples and consequently maximise the probability of detecting all the species that were present.

The study has a number of weaknesses. The sputum was not fresh when it was examined and although every effort was made to prevent contamination of samples by spores in the air, this is a possibility. The sample size is small and therefore may not be representative of asthma patients. Unfortunately, information on pets was not collected in this study and therefore could not be correlated with the presence of absence of particular fungi. It is possible to speculate that the presence of a pet (particularly a dog) in the subjects' house might be associated with the presence of *Malassezia pachydermatis* in the sputum of the research subjects, as this organism has been identified as a commensal on the skin of dogs and have could contaminated air in the homes of some of the research participants.

The potential significance of these fungi is unclear. There is tentative emerging evidence that microbiota may form part of a complex causal web that results in disease, for example, by their effects on the immune system, without becoming pathogenic in the classical sense. For example, microbial compounds present in sputum may play a role as adjuvant factors and encourage a Th2-biased allergic response [22,23].

## Conclusion

This study provides emerging evidence for the widespread presence of fungi in the sputum of asthma patients and control subjects. Significant differences have been identified in the pattern of fungi present in asthma patients and control subjects drawn from the same community. Although this method demonstrates the possibility of using microscopy samples, further investigation is warranted which applies these techniques to fresh sputum samples. This method may in itself be applicable to analysis of historical samples and may in turn prove of interest in evaluating the microbiome of the lung demographically and between generations.

## Competing interests

The authors declare that they have no competing interests.

## Authors’ contributions

HCVW obtained the initial funding, designed the case control study, obtained ethical and NHS R&D permission and wrote the first draft of the paper. CG, RG and JM undertook analysis of the sputum samples. All authors have contributed to, read and approved the final manuscript.

## Pre-publication history

The pre-publication history for this paper can be accessed here:

http://www.biomedcentral.com/1471-2334/13/69/prepub
